# Life cycle energy efficiency and environmental impact assessment of bioethanol production from sweet potato based on different production modes

**DOI:** 10.1371/journal.pone.0180685

**Published:** 2017-07-03

**Authors:** Jun Zhang, Chunrong Jia, Yi Wu, Xunfeng Xia, Beidou Xi, Lijun Wang, Youlong Zhai

**Affiliations:** 1 College of Land and Resources, China West Normal University, Nanchong, Sichuan, China; 2 Gui Zhou Academy of Environmental Science and Designing, Guiyang, Guizhou, China; 3 Chinese Research Academy of Environmental Sciences, Beijing, China; 4 College of Environmental and Municipal Engineering, Lanzhou Jiaotong University, Lanzhou, Gansu, China; University of Huddersfield, UNITED KINGDOM

## Abstract

The bioethanol is playing an increasingly important role in renewable energy in China. Based on the theory of circular economy, integration of different resources by polygeneration is one of the solutions to improve energy efficiency and to reduce environmental impact. In this study, three modes of bioethanol production were selected to evaluate the life cycle energy efficiency and environmental impact of sweet potato-based bioethanol. The results showed that, the net energy ratio was greater than 1 and the value of net energy gain was positive in the three production modes, in which the maximum value appeared in the circular economy mode (CEM). The environment emission mainly occurred to bioethanol conversion unit in the conventional production mode (CPM) and the cogeneration mode (CGM), and eutrophication potential (EP) and global warming potential (GWP) were the most significant environmental impact category. While compared with CPM and CGM, the environmental impact of CEM significantly declined due to increasing recycling, and plant cultivation unit mainly contributed to EP and GWP. And the comprehensive evaluation score of environmental impact decreased by 73.46% and 23.36%. This study showed that CEM was effective in improving energy efficiency, especially in reducing the environmental impact, and it provides a new method for bioethanol production.

## Introduction

Oil consumption in China reached 4.84 hundred million tons in 2012, with oil imports accouting for 57.1% of total consumption. China’s external dependence for oil is greater than that of America which imports 53.5% of its total consumption [1]. China’s high dependence on imported oil has raised important security concerns which need to be assessed. At the same time, the Chinese government pledged to reduce its carbon intensity emissions by 40%-45% per unit of GDP by 2020, compared with 2005 levels [2]. The development of renewable energy was a key strategey to sustain economic growth and to improve the environment [3].The Renewable Energy Act, implemented in 2006, strongly advocated the development of renewable energy, including fuel ethanol, and the National Renewable Energy Medium-Long Term Developement Plan (2007) explicityly set to increase bio-fuel ethanol yields to 10 million tons by 2020.

Bioethanol is generally considered a type of renewable energy which could lessen China’s dependency on external oil sources and effectively reduce greenhouse gases emissions [4]. Meanwhile, some investigations have indicated that bioethanol production has a positive net energy efficency and environmental benifit. For example, through the energy efficiency analysis, Dai et al.[5] concluded that the net energy and net renewalbe energy values of cassava ethanol in Guangxi were 7.475MJ/L and 7.881MJ/L, respectively. Wang et al.[6] indicated that the net energy gain of bioethanol production from sweet sorghum was 8.37 MJ/L based on life cycle analysis. Nguyen et al.[7] indicated that GHG emission of ethanol from cassava in Thailand reduced 1.6kg CO_2_ eq. per liter than gasoline. Yang et al.[8] evaluated the production system of cassava-based bioethanol by emergy analysis, which showed the transformity of 1.10×10^5^sej/J. However, some investigations have shown that biofuel has had negative energy efficency. The study of Papong et al.[9] indicated that cassava-based bioethnaol had a negative net energy value with an energy ratio was less than 1. Additionally, some researchers believe that greenhouse gas emissions will only be transferred from the bioethanol combustion stage to the production stage, or to appear in other forms such as wastewater and solid waste [10].Through life cycle assessment, the researchers concluded that the coal generated steam during bioethanol conversion unit was the major contribution to energy comsumption and CO_2_ emission [3]. Therefore, Saga et al. [11] analyzed the energy efficiency of a high-yield rice plant bioethanol production system based on the different utilizations of straw and husk, and Zhou et al. [12] integrated different energy conversion processes by poly-generation to decrease use of energy resources for bioethanol production. Laude et al. [13] considered that CO_2_ emission decreased by 115% by carbon capture and storage during ethanol production. The above studies showed that the rational utilization of by-products or wastes could effectively improve energy efficiency and environmental benefits.

The circular economy system follows the principle of mutual benefit, coexistence and resource sharing to improve resource utilization efficiency and to reduce environmental pollution. This is achieved by material recycling and energy cascade utilization to promote the sustainable development of a social, economic and environmental compound ecosystem. It is characterized as "low input, high use, low emission" [14]. Under the current production condition, the circular economy system can provide a method for production of bioethanol which reduces external energy inputs and pollution emissions. However, the recycling of substances mainly occurs in the bioethanol conversion phase, which is less considered between plant cultivation phase and bioethanol conversion phase [12, 13]. Therefore, based on current bioethanol technology, this study examines three modes of production to analyze the life cycle energy efficiency and environmental impact of sweet potato-based bioethanol production in southwest China. The study aims to (1) evaluate the energy efficiency and environmental impact of the different bioethanol production modes; and (2) compare the changes of energy efficiency and environmental impact in the different bioethanol production modes and select the optimal production mode. Through the assessment of energy efficiency and environmental impact, to identify the key drivers factors of environmental impact, and to provide reference for energy saving and emission reduction of bioethanol production.

## Materials and methods

### System boundary and functional unit

**Fig 1:**
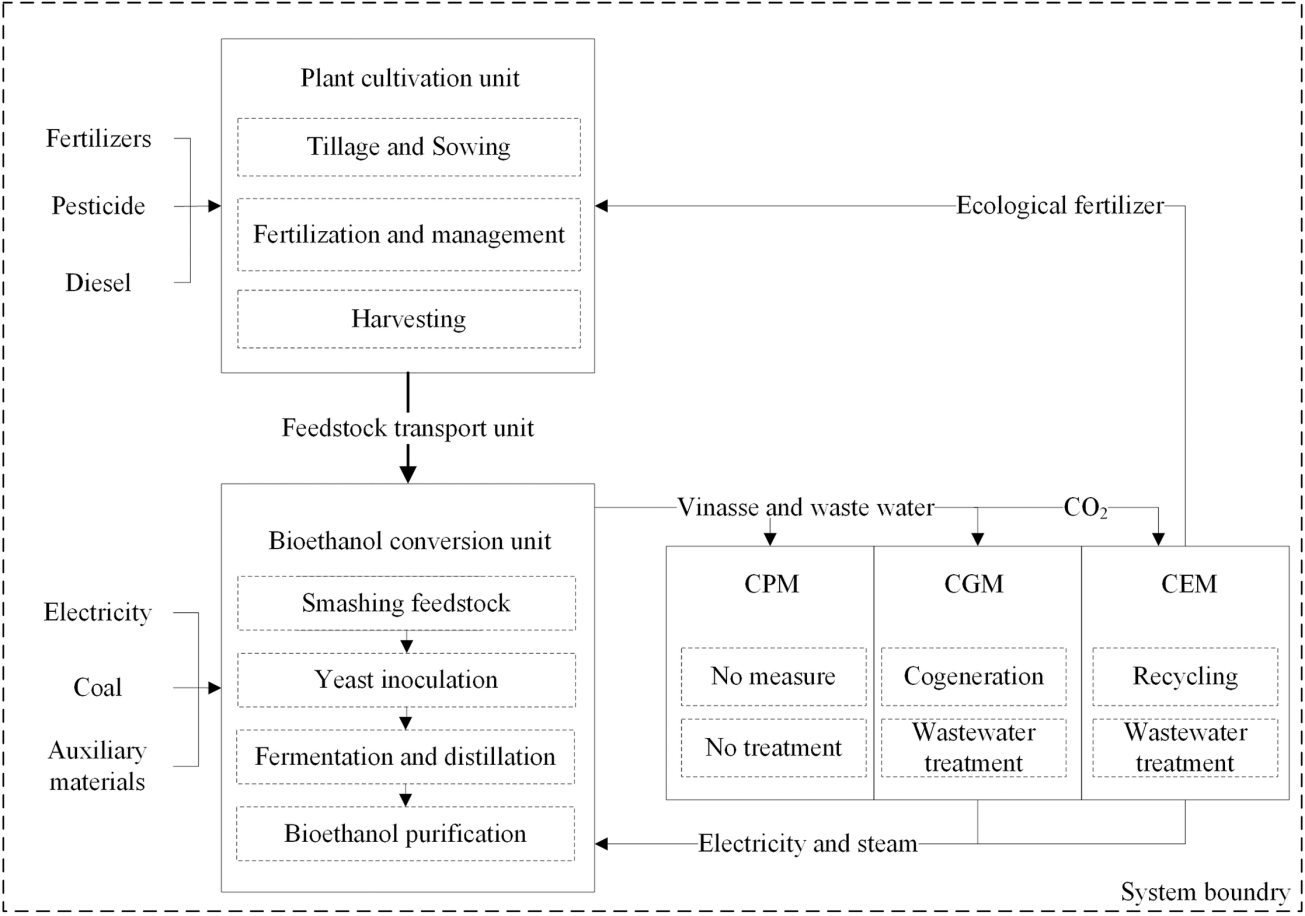
The three modes of bioethanol production and their product system boundaries. CPM, CGM and CEM represent the conventional production mode, the cogeneration mode and the circular economy mode, respectively.

The bioethanol product system boundary used in this study is presented in Fig 1. The product system includes three units: the plant cultivation unit, the feedstock transport unit and the bioethanol conversion unit. Units are linked through material flows, energy flows and service flows. Three bioethanol production modes were used to analyze utilization of by-products in the production process (Fig 1). 1. The conventional production mode (CPM) did not recycle any by-products from the production process; 2. The cogeneration mode (CGM) used electricity and heat for the bioethanol production system that was generated from biogas produced by the distillation waste (vinasse); and 3. The circular economy mode (CEM) extended the recycling of by-products from the CGM by recycling wastes such as CO_2_ and solid wastes.

The functional unit of this study is 1000L of 99.5% bioethanol production from sweet potato.

### Description of the product system

#### Plant cultivation unit

Sweet potato is an important non-staple crop which is mainly distributed in the tropical and temperate regions in China. The hilly regions of the middle and lower Yangtze River account for 60% of the total planting area in China; this area produces a yield in excess of 1×10^8^ t. For our investigation the sweet potato planting area located in southwest China, having a yield of 35t/hm^2^, was used. The plant cultivation unit included sowing, fertilization, management and harvesting etc. During the growth period, fertilizers were applied at 157.5kg N, 81kg P_2_O_5_ and 247.5kg K_2_O per hm^2^, and 1.8kg/hm^2^ of pesticides were used. Diesel was used to run small agricultural machinery. In this study, water irrigation depended on rainfall, therefore the input inventory did not include water consumption.

#### Feedstock transport unit

The feedstock transport unit was the process that transported fresh sweet potatoes from the field to the bioethanol production enterprise. In this study, seven and a half tons of fresh sweet potatoes were transported to produce 1 ton of bioethanol with a purity of 99.5%. After adopting the new business model, i.e. “Enterprise + Farmer”, the average transport distance was 30 km. Assuming vehicles must return to their starting point, the total transport distance was 60 km. This therefore included a 30 km journey with no cargo. Diesel trucks were used as the mode of transport, the consumption of diesel being 0.06L/ (km·t).

#### Bioethanol conversion unit

The conventional production mode (CPM): The CPM usually paid attention to the economic benefits and ignored the environmental benefits. It mainly included ethanol conversion enterprises, this also being the core part of all ethanol production modes. Seven and a half tons of fresh sweet potatoes were air-dried and crushed to obtain 2.78 tons of sweet potato powder. Through cooking, saccharification and fermentation 9.64 tons of fermented mash were produced and 0.75 tons CO_2_ were discharged. Subsequently, the fermented mash was distilled and purified by inputting 3.8 tons of steam. Finally, produced 1 ton of bioethanol with a purity of 99.5%, 12.44 tons distillation waste (vinasse) and a few by-products. In the ethanol production process the main inputs included steam, electricity and auxiliary materials (sulfuric acid, sodium hydroxide, yeast and amylase, etc.). Electricity was supplied by the National Grid, and it was assumed that all electricity was generated by coal combustion. Steam was produced using a coal fired steam boiler with 1kg steam being generated by 0.145kg coal [15]. During the production process, waste water, waste gas and solid waste were directly emitted; the concentration of chemical oxygen demand (COD) was up to 62530mg/L.

The cogeneration mode (CGM): In the CGM, the formation of biogas from the vinasse was collected and used to generate electricity and steam. Currently, electricity and heat production technology using biogas are advanced. In Japan, for example, the conversion rate of biogas to electricity is around 24%, and 50% for producing heat [11]. In this study, biogas used to generate electricity and steam was obtained by anaerobic treatment of solid residue that had been separated from the vinasse. From the components of the biogas, methane accounted for 55% of the total biogas. The electric energy conversion rate using biogas was 1.3–1.4kwh/m^3^ CH_4_; for the purposes of this study a value of 1.35kwh/m^3^ CH_4_ was used. Sewage (11.84t) separated from the vinasse was treated using internal circulation (IC) and an upflow anaerobic sludge bed (UASB) reactor. The wastewater from this treatment meets the National Standard before being discharged into local water courses. For simplicity, energy input of the sewage treatment plant was replaced by electric energy; 0.29kwh energy is needed to process 1 m^3^ of sewage in China [16].

The circular economy mode (CEM): The general principle of the CEM was to save energy and reduce emissions by recycling the waste products. For this study, two circular production chains were established on the basis of CGM: (1) CO_2_ emitted from the distillation process was collected by washing, compressing, purifying, drying and condensing to produce liquid CO_2_ for sale; and (2) after cogeneration and sewage treatment, the biogas residue and the anaerobic sludge were used to produce bio-fertilizer (0.36t) which was used as a substitute for fertilizer currently used in the production process.

### Life cycle inventory (LCI)

LCI was performed on the sweet potato bioethanol production process. The input and output data related to materials and energy were predominantly derived from the enterprise investigation and interviews with farmers during the plant cultivation unit, the feedstock transport unit, and the bioethanol conversion unit. The emission data related to fossil fuel combustion (coal and diesel) and auxiliary materials (fertilizers, pesticides, yeast, amylase, H_2_SO_4_, NaOH and electricity) were mainly derived from the GREET model that was developed by Argonne National Laboratory (ANL, 2012). In this study, according to the China’s conditions we calibrated the parameters in the model, such as energy structure and efficiency, etc. Soil emissions derived from application of fertilizer and runoff loss were estimated in term of the studies of Wang and Zhang et al. [17, 18]. The emission factors of pesticide applied were referenced from Räsänen et al. [19]. Electricity mainly derived from coal combustion in China, so the emission factors of electricity and coal production were estimated based on the results of the study by Leng et al. [20]. The emission factors of biogas combustion were referenced from Chen and Jury et al. [21, 22].

Table 1 shows the primary fossil energy input and emissions inventory based on the production of 1000L of 99.5% sweet potato bioethanol. Microsoft Excel 2013 was used to process the inventory data.

**Table 1:** Life cycle inventory for 1000L bioethanol produced from sweet potato using the difference modes.

Emissions	CPM	CGM	CEM
Primary fossil energy input			
Coal (kg)	690.18	361.09	357.14
Crude oil (kg)	33.96	33.96	33.96
Emissions			
CO (g)	1297.34	802.81	809.84
N_2_O (g)	484.43	483.40	368.23
CO_2_ (kg)	1320.98	1120.31	383.26
CH_4_ (g)	962.53	678.75	661.38
SO_2_ (g)	2728.18	1400.82	1675.91
NO_x_ (g)	2376.21	1530.74	1585.49
NH_3_ (g)	3221.99	3221.99	2453.42
PM10 (g)	291.98	130.99	149.99
VOC (g)	122.38	129.82	140.42
TN (g)	3900.00	3900.00	2676.72
TP (g)	282.80	282.80	238.13
COD(kg)	740.77	0.41	0.77
Pesticide to air (g)	21.76	21.76	21.76
Pesticide to water (g)	2.18	2.18	2.18
Pesticide to soil (g)	193.69	193.69	193.69

### Energy efficiency analysis

Net energy ratio (NER) and net energy gain (NEG) are two important indicators for evaluating life cycle energy inputs and outputs. The calculation formulae are:

$$NER=E_{out}/E_{in}$$ (1)

$$NEG=E_{out}-E_{in}$$ (2)

where, *E*_*out*_ is energy output, taken as 23.27MJ/L [6] as the lower heating value of the bioethanol; and *E*_*in*_ is the total amount of energy inputs required to produce 1L of bioethanol, which is calculated according to the following equation:

$$E_{in}=\sum M_{i}\times c_{i},i=1,\ldots ,n$$ (3)

Where *M*_*i*_ is the *i*th input matter flow and *c*_*i*_ is corresponding energy intensity, which is obtained from literature [9, 11, 23].

### Impact assessment of life cycle

Bioethanol production from sweet potatoes creates three types of environmental emissions: waste gas, wastewater and solid waste. In order to compare the environmental impact of the emissions, the life cycle of the environmental emissions were characterized, standardized and weight evaluated in this study.

Characterization of the emissions involves classifying the environmental emissions and calculating the potential contribution of emissions to various potential impacts. According to the method of CML-IA 2000 (Centre of Environmental Science), potential environmental impact related to the life cycle of sweet potato bioethanol production were divided into five categories: global warming potential (GWP), photochemical oxidation potential (POCP), acidification potential (AP), eutrophication potential (EP), and human toxicity potential (HTP). These categories were generally expressed using CO_2_ eq. (over a 100-year period), C_2_H_4_ eq., SO_2_ eq., ${\text{PO}}_{4}^{3-}$ eq. and 1,4-DCB (1,4 dichlorobenzene), respectively. Characterization factors for all pollutions were obtained from the CML-IA 2000 database.

Standardization provides a method for comparing the various environmental impact categories and broadens the context of the LCIA indicator results. In standardization, it provided a reference system by using the overall indicator results for a specific region, such as a city, a country or whole world. The scores of five environmental impact categories were calculated using the standardization factors derived from Sleeswijk [24]. The score was standardized by dividing the environmental impact potential with the average environmental impact potential per world capita in 2000 [6].

Weighted evaluation is a method of comprehensive evaluation which aids in calculating the environmental impact comprehensive score based on the weight of each category’s potential environmental impact. This method also compares the advantages and disadvantages of the different bioethanol production systems. The weight coefficients derive from the findings of Wang [25].

## Results and discussion

### Energy efficiency

Table 2 shows the results of the energy input–output of sweet potato-based bioethanol production. Results for NER were 1.23 (CPM), 2.20 (CGM) and 2.23 (CEM); NEG results were 4.37 (CPM), 12.71 (CGM) and 12.81 (CEM) MJ/L. This indicated that energy output was greater than input, and that the NER and NEG results for CEM were higher than the other modes under the current technical conditions. The energy input of the plant cultivation unit accounted for 19.26–34.47% of the total input, whilst the feedstock transport unit accounted for a very small amount of the total input. The energy input of the bioethanol conversion unit (58.99–77.09%) accounted for the greatest energy use in the bioethanol production system; electricity and steam generation using coal was the main energy use. This result was similar to other studies [9, 26]. The energy input of the bioethanol conservation units in CGM and CEM, utilizing cogeneration to reduce the production of electricity and steam by coal, were reduced by 57.24% and 53.74%, respectively, in comparison with CPM. Saga et al. [11] believed that the energy efficiency of a bioethanol production system was improved by cogeneration. The energy input of CEM increased by 8.19% in comparison with CGM, mainly due to increases in the production chain with this mode. However, in the plant cultivation unit, CPM and CGM did not implement control measures on energy input while CEM replaced a proportion of traditional fertilizers with bio-fertilizers. This resulted in an energy input reduction of 20.53%.

**Table 2:** Primary energy input-output and energy efficiency of bioethanol produced from sweet potato for the different modes.

	Processes	CPM	CGM	CEM
*E*_*in*_ (MJ/L)	Plant cultivation unit	3.64	3.64	3.02
	Nitrogen	2.14	2.14	1.63
	Phosphorus	0.20	0.20	0.17
	Potassium	0.42	0.42	0.35
	Pesticides	0.15	0.15	0.15
	Diesel	0.72	0.72	0.72
	Feedstock transport unit	0.70	0.70	0.70
	Bioethanol conversion unit	14.57	6.23	6.74
	Electricity	1.75	0.47	0.98
	Auxiliary materials	0.11	0.11	0.11
	Coal	12.70	5.65	5.65
	Total fossil energy input	18.90	10.56	10.46
*E*_*out*_ (MJ/L)		23.27	23.27	23.27
NER		1.23	2.20	2.23
NEG(MJ/L)		4.37	12.71	12.81

### Environmental impact analysis

**Fig 2:**
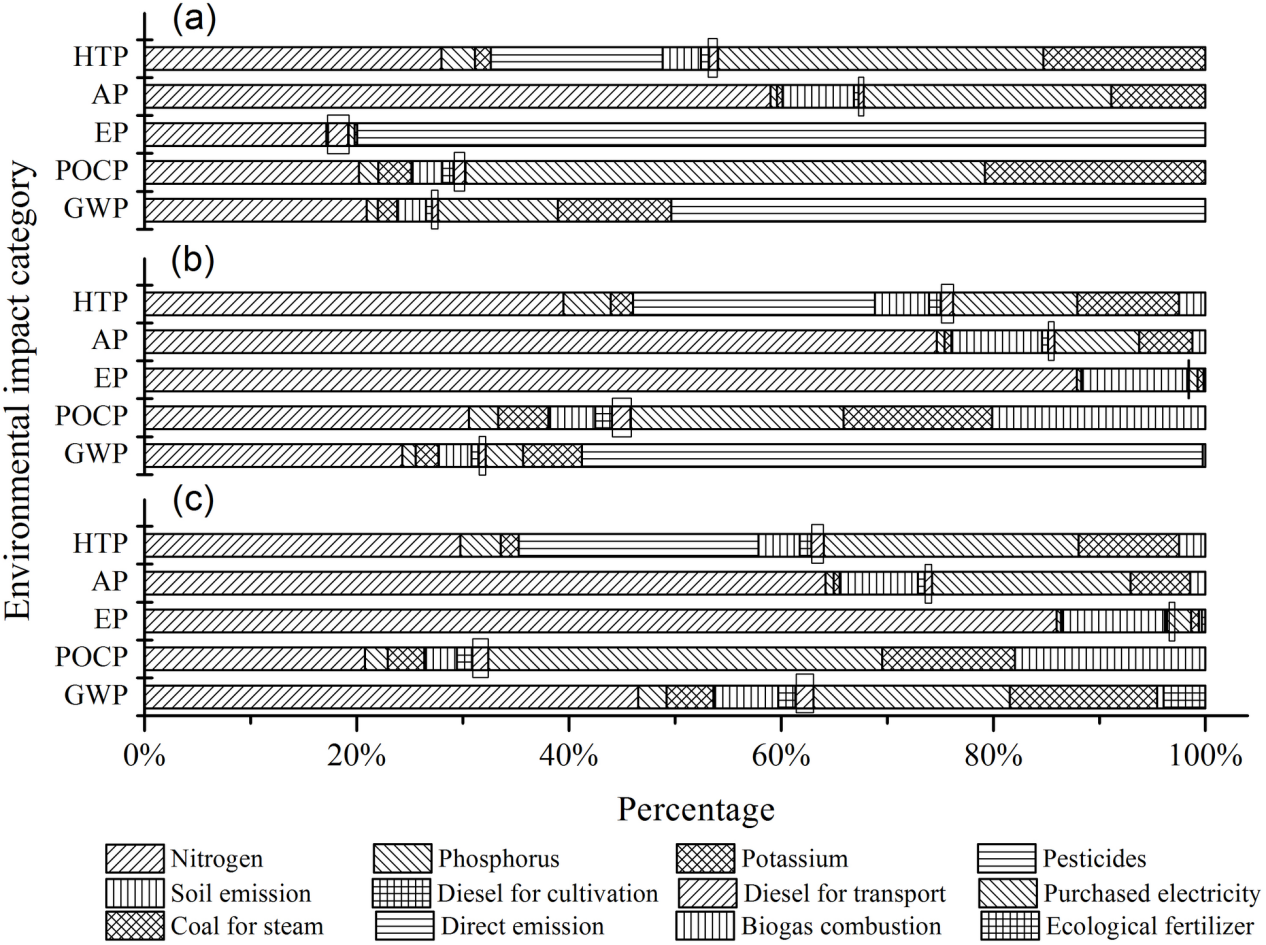
Environmental impacts of bioethanol production process in CPM (a), CGM (b) and CEM (c). Results in the squares indicate the environmental impact of the feedstock transport unit, results to the left of the squares are the environmental impact of plant cultivation units, and results to the right are the environmental impact of the bioethanol conversion unit.

The environmental impact categories for the three production modes are shown in Fig 2 and Table 3. The environmental impacts of AP and HTP for CPM were greatest in the plant cultivation unit; the environmental impacts of GWP, POCP and EP were dominant in the bioethanol conversion unit. For CGM and CEM, the environmental impact categories were more dominant in the plant cultivation unit than in the bioethanol conversion unit, except for GWP in CGM and POCP in CEM. The potential environmental impact in the feedstock transport unit was small for all three modes, thus our discussion mainly focuses on the potential environmental impact of the plant cultivation unit and the bioethanol conversion unit.

**Table 3:** Environmental impact potential of 1000L bioethanol produced from sweet potato for the three modes.

Environmental impact category	Unit	CPM	CGM	CEM
GWP	kg CO_2_ eq.	1489.40	1281.33	509.52
POCP	kg C_2_H_4_ eq.	0.22	0.15	0.16
EP	kg PO_4_ eq.	20.37	3.97	3.03
AP	kg SO_2_ eq.	9.62	7.60	6.73
HTP	kg 1,4 DCB eq.	4.38	3.11	3.14

#### Global warming potential (GWP)

The bioethanol conversion unit was the main environmental impact source for GWP in CPM and CGM, accounting for 72.34% and 67.85% of bioethanol production, respectively. CO_2_ directly emitted from the distillation process (including saccharification and fermentation) was the major sources of GWP, contributing 50.36% and 58.53% of GWP, respectively. For CEM, CO_2_ was recycled so that GWP in the bioethanol conversion unit decreased by 84.38% compared with CPM and 80.65% compared with CGM. This showed that recycling CO_2_ could significantly reduce the GWP of the production process. Research by Laude et al. [13] has also shown that greenhouse gas emissions can be significantly reduced by capturing CO_2_ in the bioethanol conversion unit. Additionally, fertilizer production was another important environmental impact source for GWP in all modes, the percentage of GWP ranged from 20.91% to 46.53%.

#### Eutrophication potential (EP)

For CPM, the EP predominantly derived from the bioethanol conversion unit accounted for 80.58% of the total EP. The main source of the EP was the release of organic compounds in the wastewater which increased the COD of the local water courses. This pollution accounted for 79.77% of the total EP, equating to about 16.29 kg ${\text{PO}}_{4}^{3-}$ eq. Xia et al. [27] proposed that the main cause of eutrophication was due to untreated wastewater from the bioethanol production system. After treatment measures were implemented for the wastewater, EP of the bioethanol conversion unit in CGM and CEM decreased to 0.06 kg ${\text{PO}}_{4}^{3-}$ eq. and 0.09 kg ${\text{PO}}_{4}^{3-}$ eq., accounting for 1.59% and 3.28% of the total EP, respectively. The main contribution of the EP was transferred from the bioethanol conversion unit to the plant cultivation unit. Consequently, it is important to reduce the EP by treating wastewater during the production of bioethanol.

#### Acidification potential (AP)

The plant cultivation unit was the major contributor to the AP in all modes, the percentage of the AP ranged from 67.3% to 85.15% (Fig 2). NH_3_ volatilization and nitrogen loss by runoff and leaching were the major sources of the AP during planting; this was closely related to large-scale inputs of fertilizer in agricultural production in China [28]. Additionally, in the bioethanol conversion unit, electricity and steam generated by coal was the other important contributor, accounting for 32.20%, 14.23% and 25.75% of the total AP for CPM, CGM and CEM, respectively. Through the process of cogeneration, AP decreased from 3.09 kg SO_2_ eq. (CPM) to 1.08 kg SO_2_ eq. (CGM) and 1.73 kg SO_2_ eq. (CEM). In CEM, a proportion of the fertilizer was replaced by bio-fertilizer which resulted in a reduction of 21.53% for the AP caused by fertilizers.

#### Photochemical oxidation potential (POCP)

The percentage of POCP for CPM, CGM and CEM was higher in the bioethanol conversion unit, accounting for 69.77%, 54.2% and 67.59%, respectively. SO_2_ emitted from coal combustion was the major source of POCP. This result was consistent with results by Wang [3]. Cogeneration used in CGM and CEM, POCP derived from coal generated electricity and steam were 50.09g C_2_H_4_ eq. and 81.17 C_2_H_4_ eq., reduced 67.75% and 47.38% compared with CPM, respectively. The decrease in POCP in CEM was less than that recorded in CGM as CEM incorporated recycling which was more energy dependent.

#### Human toxicity potential (HTP)

HTP was related to NO_x_, SO_2_, PM_10_ and pesticides. The results showed that the application of fertilizers and pesticides in the plant cultivation unit, combined with the consumption of fossil fuels in the bioethanol conversion unit, were the main sources of HTP. Cogeneration reduced the input of energy from fossil fuels which resulted in a reduction in HTP from 2.01 kg 1, 4-DCB eq. (CPM) to 0.66 kg 1, 4-DCB eq. (CGM) and 1.05 kg 1, 4-DCB eq. (CEM). In CEM, a proportion of the fertilizer was replaced by bio-fertilizer which resulted in HTP to decrease by 15.39% compared with the other two modes. The results for CGM and CEM indicate that cogeneration and recycling effectively reduced HTP in the bioethanol production unit and the plant cultivation unit.

### Environmental performance

**Fig 3:**
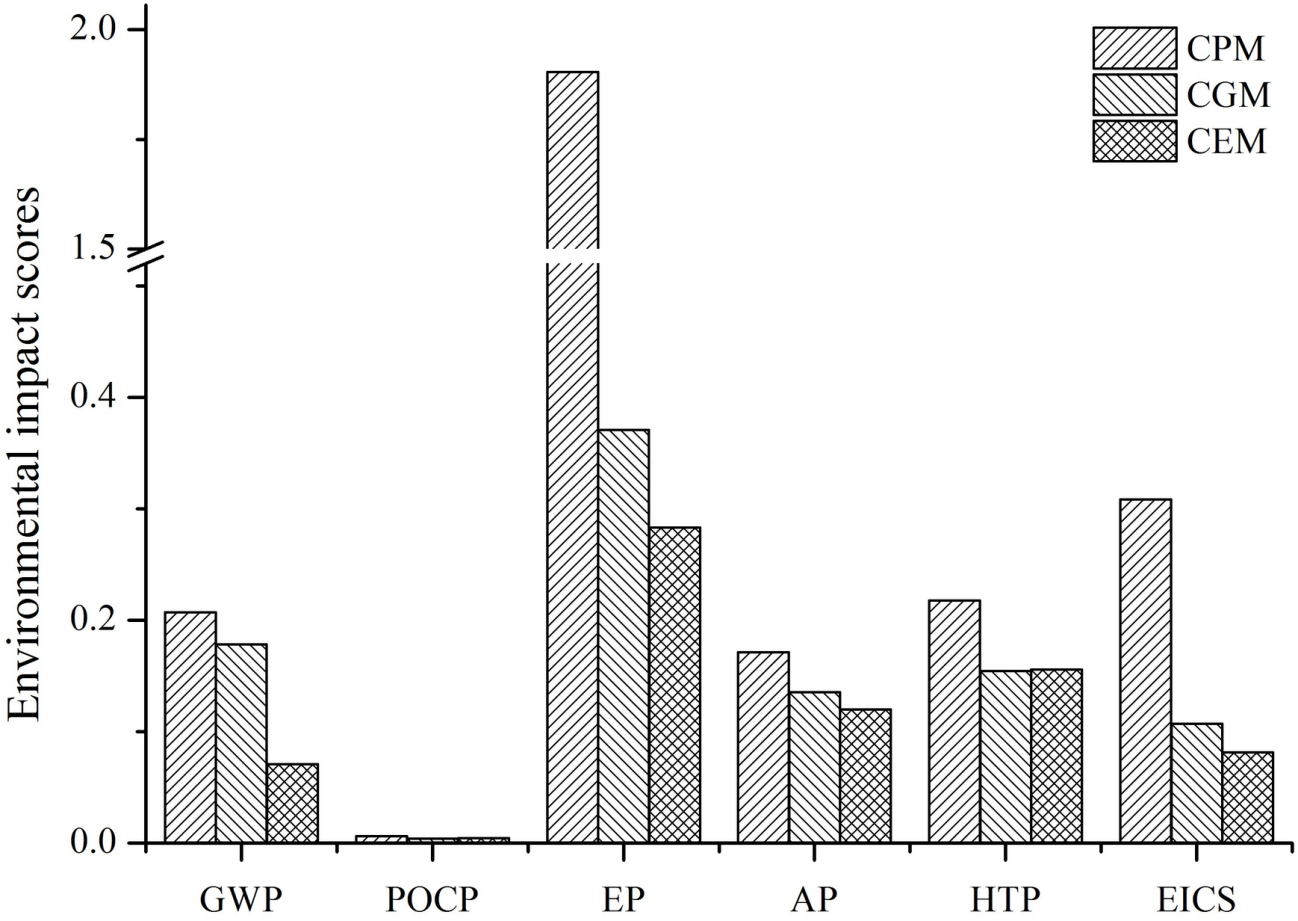
The environmental impact scores for the different production modes.

Based on the world’s per capita environmental impact potential in the year 2000, the environmental impact scores for the five environmental impact categories are shown Fig 3. For CPM, EP is the most important negative environmental impact category which has an environmental impact score of 1.903. This means that eutrophication caused by 1000L sweet potato bioethanol production was 1.903 times more than the world’s per capita in 2000. The next most important were HTP (0.218), GWP (0.207), AP (0.171) and POCP (0.006). The environmental impact scores of the different categories for CGM and CEM were all lower than those for CPM.

For CPM, the high EP result is due to the direct emission of wastewater from the bioethanol conversion unit. In CGM and CEM, due to the implementation of wastewater treatment measures, the EP score reduced to 0.371 and 0.283, respectively. Despite these changes between CPM, CGM and CEM, EP was still the most important negative environmental impact category. This was mainly caused by the production of fertilizers and the plant cultivation unit which resulted in a loss of nutrients. Thus, for the bioethanol production system, it is important to reduce EP to improve the environmental impact.

Cogeneration applied to CGM resulted in a reduction of the HTP score due to a reduction of SO_2_ and NO_x_ emissions as the use of fossil fuels reduced. Additionally, GWP also decreased as CO_2_ emissions also declined with cogeneration. This decrease however was not significant because CO_2_ linked to coal combustion only accounts for 10.12% of the total emissions. Therefore, the HTP score in CGM was lower than the score for GWP, this result being opposite to that for CPM. In CEM, CO_2_ emitted from the distillation process was recycled. This reduction, coupled with the increase of bio-fertilizers and a reduction in the use of traditional fertilizers, significantly reduced the score of GWP to 0.071.

Using the weighted evaluation, the environmental impact comprehensive score (EICS) was 0.309, 0.107 and 0.082 for CPM, CGM and CEM, respectively. The results show that CEM has the lowest environmental impact.

## Conclusions

This study analyzed the energy efficiency and environment impact of three ethanol production modes using the life cycle assessment method. The results showed that bioethanol production from sweet potato in CEM had higher energy efficiency and lower environmental impact than in CPM and CGM. For three ethanol production modes, the NEG show positive, but which increased by 193.14% and 0.85% in CEM than in CPM and CGM, respectively. The electricity and steam generation using coal was the main energy use. For CPM, the main environmental impact category was EP, which comes from the direct emission of vinasse in the bioethanol conversion unit. Additionally, HTP, GWP and POCP predominantly derived from coal combustion for steam generation and CO_2_ directly emission in the distillation process. However, for CEM, the soil nutrient losses in the cultivation unit were main contribution to EP, HTP and GWP. Compared with CPM and CGM, the comprehensive evaluation score of environmental impact in CEM decreased by 73.46% and 23.36%. So, the CEM provided a good development mode for bioethanol production because of improving energy efficiency and controlling environmental emissions. In the next step, it is a key work to improve the energy efficiency and environmental benefits during the cultivation unit.

## Supporting information

S1 Fig Basic mass balance of bioethanol process for this system. (TIF)

S1 Table Datasets for Fig 2 including environmental impacts of bioethanol production process in CPM (a), CGM (b) and CEM (c).
(XLSX)

S2 Table Datasets for Fig 3 including the environmental impact scores for the different production modes. (XLSX)
