# Supplementary figures and images for: Life cycle energy efficiency and environmental impact assessment of bioethanol production from sweet potato based on different production modes
Source: PLoS One. 2017 Jul 3;12(7):e0180685. doi: 10.1371/journal.pone.0180685 (PMC5495513; doi:10.1371/journal.pone.0180685)

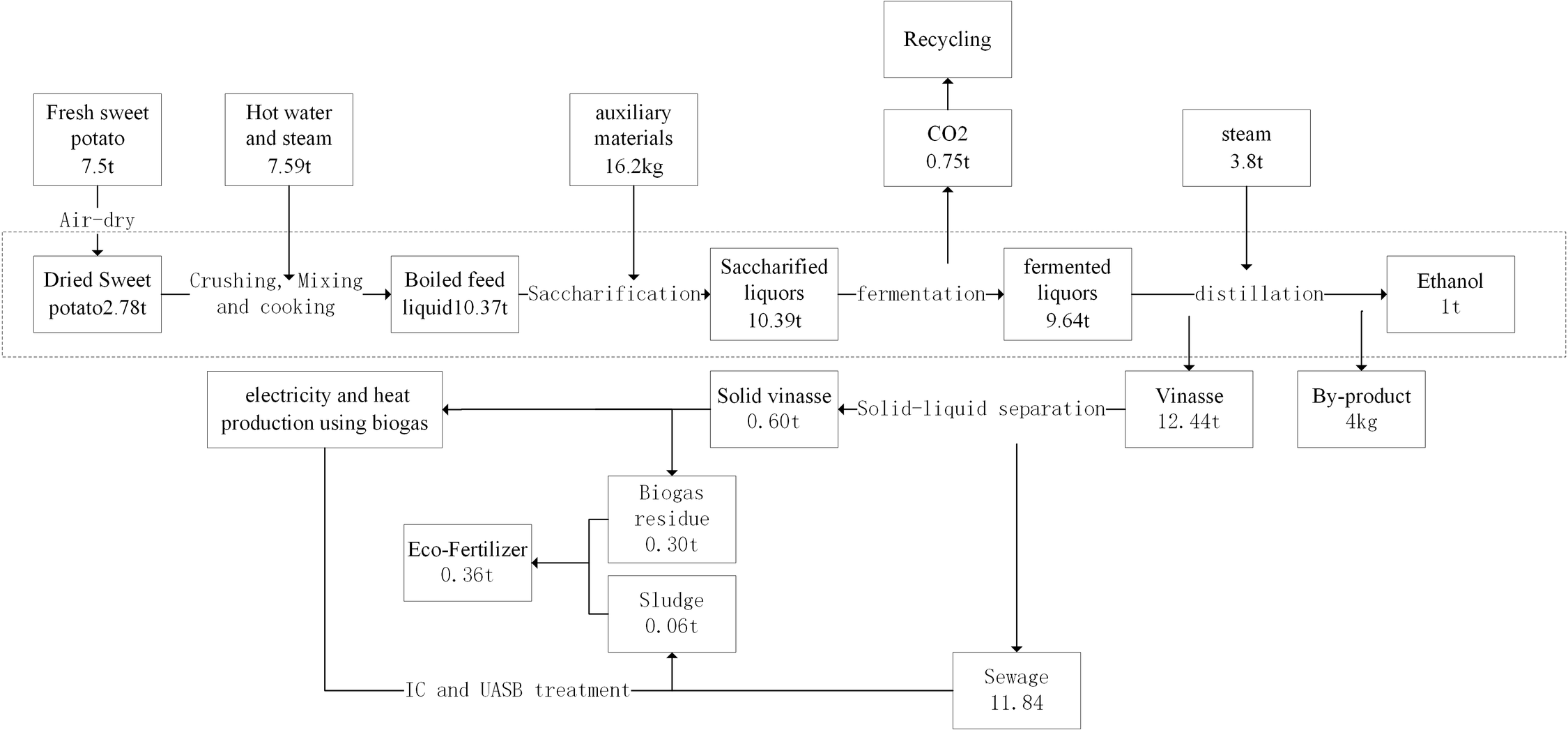

Supplement: S1 Fig — (TIF) [file pone.0180685.s001.tif]
